# Removal of Fluoride from Drinking Water by Sorption Using Diatomite Modified with Aluminum Hydroxide

**DOI:** 10.1155/2019/4831926

**Published:** 2019-12-03

**Authors:** Tesfaye Akafu, Achalu Chimdi, Kefyalew Gomoro

**Affiliations:** ^1^Wollega University, College of Natural and Computational Sciences, Department of Environmental Sciences, P.O. Box: 395, Nekemte, Ethiopia; ^2^Ambo University, College of Agriculture and Veterinary Sciences, Department of Natural Resource Management, P.O. Box: 19, Ambo, Ethiopia; ^3^Wollega University, College of Natural and Computational Sciences, Department of Chemistry, P.O. Box: 395, Nekemte, Ethiopia

## Abstract

Exposure to fluoride beyond the recommended level for longer duration causes both dental and skeletal fluorosis. Thus, the development of cost-effective, locally available, and environmentally benign adsorbents for fluoride removal from contaminated water sources is absolutely required. In the present study, diatomaceous earth (diatomite) locally available in Ethiopia, modified by treating it with an aluminum hydroxide solution, was used as an adsorbent for fluoride removal from aqueous solutions. Adsorption experiments were carried out by using batch contact method. The adsorbent was characterized using FT-IR spectroscopy. Effects of different parameters affecting efficiency of fluoride removal such as adsorbent dose, contact time, initial fluoride concentration, and pH were investigated and optimized. The optimum adsorbent dose, contact time, initial fluoride concentration, and pH values were 25 g/L, 180 min, 10 mg/L, and 6.7, respectively. The performance of the adsorbent was also tested under optimum conditions using groundwater samples taken from Hawassa and Ziway. Langmuir and Freundlich isotherm models were applied to describe the equilibrium data. Compared to Langmuir isotherm (*R*^2^ = 0.888), the Freundlich isotherm (*R*^2^ = 0.985) model was better fitted to describe the adsorption characteristics of fluoride on Al-diatomite. The Langmuir maximum adsorption capacity was 1.67 mg/g. The pseudosecond-order model was found to be more suitable than the pseudofirst-order to describe the adsorption kinetics. The low correlation coefficient value of *R*^2^ = 0.596 for the intraparticle diffusion model indicates that the intraparticle diffusion model does not apply to the present studied adsorption system. The maximum fluoride removal was observed to be 89.4% under the optimum conditions which indicated that aluminum hydroxide-modified diatomite can be used as efficient, cheap, and ecofriendly adsorbents for the removal of fluoride from contaminated water.

## 1. Introduction

Water is a source of life, a fundamental requirement for health and main need for industrialization. It is essential for all forms of growth and development: humans, animals, and plants. However, the suitability of water for a specific purpose can be affected by other substances dissolved or suspended in it. Contamination of drinking water by fluoride is one such example. In addition to arsenic and nitrate, which cause large-scale health problems, fluoride is classified as one of the contaminants of water for human consumption by the World Health Organization (WHO) [1].

Fluoride contamination of groundwater by natural as well as anthropogenic sources is a major problem worldwide, imposing a serious threat to human health. Water contamination by fluoride from industrial activities includes effluent discharge, fertilizers and pesticides, fluorosilicone and fluorocarbon polymer synthesis, coke manufacturing, glass and ceramic manufacturing, electronics manufacturing, electroplating operations, steel and aluminum manufacturing, metal etching (with hydrofluoric acid), and wood preservatives [2]. There are many ores, minerals, and rocks present inside the earth's crust, which are the natural sources of fluoride. The major sources of fluoride in groundwater are weathering and geochemical dissolution of fluoride-bearing rocks such as sellaite (MgF_2_), fluorspar (CaF_2_), cryolite (Na_3_AlF_6_), and fluorapatite (calcium fluorophosphates, (Ca_5_(PO_4_)_3_F). Because of the long contact time of fluoride-bearing ores, minerals, and rocks with groundwater, there is a constant leaching of fluoride ions that is responsible for the high fluoride concentration in groundwater as well as oceanic water [3].

Fluoride enters into the human body through a variety of sources like water, food, air, medicine, and cosmetics. Among these, drinking water is the most common source which makes fluoride available to human beings [4]. Fluoride is known to have both beneficial and detrimental effects on health, depending on the dose and duration of exposure. The optimum fluoride level in drinking water should be below 1.5 mg/L [5]. Low amount of fluoride is necessary in the prevention of tooth decay and the development of proper bone structure in humans and animals. It is considered to be a micronutrient for humans since it prevents dental caries by decreasing the rate of demineralisation of the dental enamel or reverses the progression of existing decay by promoting the rate of remineralisation. High doses of fluoride lead to the development of dental and skeletal fluorosis, depending on the concentration of fluoride in drinking water [2, 6]. Thus, fluoridation units should be established at drinking water treatment plants if the fluoride concentration is less than the desired quantity, and extra amount of fluoride must be removed from the water using appropriate methods if the fluoride concentration exceeds the permissible value.

Dental fluorosis is the most common manifestation of chronic use of high-fluoride water and is characterized by discoloured, blackened, mottled, or chalky-white teeth. Skeletal fluorosis occurs over long-time consumption of drinking water with >4 mg/L fluoride during adolescence which may disrupt the mineralization of bones leading to severe and permanent bone and joint deformations [7]. Fluorosis not only affects the body of persons but also renders them socially and culturally crippled [8]. For instance, younger people showing symptoms of high dental fluorosis (severe pitting and coloration of teeth) find it difficult to smile comfortably in public.

Fluoride contamination of groundwater and related health hazards are a worldwide problem, including various countries in Africa, Asia, and Europe as well as the USA and Australia. China and India are among the most affected, and other countries such as Ethiopia, Kenya, Ghana, and Tanzania have serious problems related to fluoride contamination [9]. Hence, it is estimated that more than 260 million people worldwide are affected by fluorosis [5]. Most endemic fluorosis occurs in rural areas of developing countries, where the less-developed economy and technological challenges aggravate the problem of unsafe drinking water. Fluoride concentration above 1.5 mg/L has been reported from many parts of Ethiopia, but the highest levels are found in the Rift Valley, the lowland area with the highest volcanic activity in the country. In the Ethiopian Rift Valley, about 14 million people rely on water sources that contain high concentrations (above 5 mg/L) of fluoride. Therefore, an economical and effective method for fluoride removal in drinking water is of great importance [10].

Fluorosis is a disease for which no medical treatment exists and considered as crippling disease, and prevention is the only solution for this menacing problem. Fluoride poisoning (fluorosis) can be prevented or minimized by using alternative water sources (like surface water, low-fluoride groundwater, and rain water), increasing the nutritional status of the population at risk (adequate intake of calcium reduces the risk of dental fluorosis during the childhood), and by removing excessive fluoride from drinking water. Defluoridation of drinking water appears to be a simpler practical solution to prevent the adverse effects of fluoride. Hence, the development of defluoridation technologies, preferably low-cost and environmentally friendly, capable of reducing the fluoride concentration below the limit established by the WHO is of paramount importance [7].

Different technologies have been used for the removal of fluoride from drinking water including precipitation/coagulation, membrane-based processes, ion-exchange methods, and adsorption methods. Lime and alum are used to form insoluble precipitates with fluoride in precipitation and flocculation process. The fluoride concentration of the treated solution still remains about 8.0 mg/L due to the solubility of the formed precipitate. Chemical precipitation may also produce large amount of sludge [7, 9–11]. Membrane separation process mainly contains reverse osmosis and nanofiltration. Even though it can produce water with extremely high purity, it suffers from high energy consumption and membrane fouling. In electrodialysis process, fluoride ions transfer through a semipermeable membrane due to an electric potential. It is costly and easily influenced by coexisting ions [1]. Most of the available defluoridation techniques are complex, require skilled labor, and have a high initial and maintenance cost and technically nonfeasible for rural areas. Hence, the need to find locally available defluoridation media for safe and easy use at both household and small community levels is desirable [3–6].

Adsorption is the preferred technique for defluoridation at community and household levels in rural areas because of its low cost and ease of operation, high efficiency, easy accessibility, environmental benignity, and needless of operational skill and electric power to run, and since adsorbents can in principle be reused and recycled making it ideal for use in less-developed rural areas. It has the added advantage that it can be applied to a decentralized water supply system. The availability of different adsorbents in large amounts and low costs make them potential candidates for the defluoridation in remote areas [2–5].

Diatomites (diatomaceous earth) are siliceous sedimentary rocks composed mainly of amorphous hydrated or opaline silica (SiO_2_·*n*H_2_O) with varying amounts of impurities such as clay minerals, silica sand, carbonate minerals, iron oxides, and organic matter. Diatomaceous earth (DE) is of particular interest as an adsorbent due to its unique properties such as high porosity (typically 10–200 *µ*m), high permeability, small particle size, high surface area, low thermal conductivity, and chemical inertness [12]. DE is nonorganic and so cannot undergo degradation to foul water [13]. In addition, diatomite is approximately 500 times cheaper than commercial activated carbon and has the potential of being used as a cost-effective alternative to activated carbon. The chemical composition and physical structure of diatomite give it great commercial value for a wide range of applications, including beer filter aids, the removal of textile dyes from wastewater, uptake of different water contaminates, and the sorption of heavy metal ions [14].

Despite the unique combination of physical and chemical properties of diatomite and its abundant availability in Ethiopia, its application for fluoride removal from drinking water in Ethiopia has not been investigated properly. The effectiveness of adsorption techniques is greatly dependent on the physicochemical properties of the adsorbent. Raw diatomaceous earth (DE) has a low fluoride removal potential. The highest percent fluoride removal at optimum adsorption conditions is between 23.4% and 25.6% for 8 mg/L fluoride at pH 2, contact time of 30 min, solid-liquid ratio of 0.4 g/50 mL, and shaking speed of 200 rpm [13]. Thus, surface modification of DE so that it can have high fluoride adsorption capacity is necessary. Therefore, the main aim of this study is to investigate the effectiveness and efficiency of fluoride ion-sportive removal by diatomite modified with aluminum hydroxide from aqueous solutions.

## 2. Materials and Methods

### 2.1. Preparation of Stock and Standard Solutions

A 1000 mg/L stock solution of sodium fluoride (NaF) was prepared by dissolving 2.21 g of anhydrous sodium fluoride (99.0% NaF, BDH Chemicals Ltd, England) in distilled water in a 1 L volumetric flask and diluting to the mark. Other standard fluoride solutions of the required concentrations for calibrating the fluoride ion-selective electrode (FISE) were prepared by serial dilution of the stock solution with distilled water.

### 2.2. Preparation of Total Ionic Strength Adjustment Buffer

Calibration and determination were carried out by addition of total ionic strength adjustment buffer (TISAB). The TISAB was prepared following a recommended procedure by dissolving 57 mL glacial acetic acid (100%, Sigma-Aldrich Laborchemikalien, Germany), 58 g sodium chloride (Oxford Laboratory, Mumbai, India), 7 g of sodium citrate (BDH Chemicals, England), and 2 g of EDTA (Scharlau Chemie S. A., Barcelona, Spain) in 500 mL distilled water; and then the pH was adjusted to 5.3 with 5 M sodium hydroxide (Scharlau Chemie S. A., Barcelona, Spain) and then made up to 1000 mL in a volumetric flask with distilled water [15].

### 2.3. Adsorbent (Diatomite) Preparation

The DE used for the study was collected in polyethylene plastic bags from Bedele Brewery, Oromia Regional State, Ethiopia. The material was washed with distilled water to remove dirt and dried under sun. The coating of aluminum hydroxide onto diatomite was carried out according to the method used by Wang and Peng [16] with slight modification. 30 g of powdered diatomite was taken in a 1 L plastic bottle, and 100 mL of 1 mol/L AlCl_3_·6H_2_O and a certain amount of 3 mol/L NaOH was immediately added into the mixture. The mixture was shaken at room temperature for 2 hours on a shaker at 200 rpm. The mixture was centrifuged after equilibration, and the recovered solid was scooped into a 1 L bottle containing some distilled water. After acidifying the mixture with 0.1 M HCl to pH 2, it was shaken for 40 min and centrifuged. The solid was washed with distilled water with repeated centrifuging until the pH of the supernatant was 6. The solid residues were dried in the oven at 105°C for 12 h, cooled in the desiccator, and sieved through an 80 mesh sieve. The dry sorbent was stored in a corked plastic bottle until use.

### 2.4. Apparatus and Instruments

An electronic balance (Adam Equipment, Model WL 3000, UK), with precision of 0.0001 g was used for weighing adsorbents and chemicals for solution preparation. An oven (Digit heat, J. P. Selecta, Spain) was used for drying the adsorbent and glass wares during analysis. A pH meter (HANNA instrument, HI 9025, Singapore) equipped with a pH glass electrode was used to measure the pH values of sample solutions. A pH/ISE meter (Orion model, EA 940 Expandable Ion Analyzer, USA) equipped with a combination fluoride ion-selective electrode (Orion Model 96-09, USA) was employed for the determination of fluoride in the samples and standards solutions. Spectrum 65 FT-IR (Spectrum 65 FT-IR spectrometer (PerkinElmer, USA) was used to record IR spectra.

### 2.5. FT-IR Analysis

To evaluate the likely changes in the functional groups of the material in contact with fluoride solution, the FT-IR spectroscopic analyses of both raw DE and aluminum hydroxide-treated DE were done using a Spectrum 65 FT-IR spectrometer (PerkinElmer, USA) in the KBr pellet in the range 4000–400 cm^−1^.

### 2.6. Fluoride Adsorption Studies

The bath adsorption and defluoridation studies were conducted in order to optimize various experimental parameters like contact time, initial fluoride concentration, adsorbent dose, and pH which can affect the adsorption efficiency of fluoride onto DE. Batch mode adsorption studies were carried out by agitating 25 g/L of the adsorbent in 50 mL of 10 mg/L ﬂuoride solutions at pH 6.7 for 180 min taken into 250 mL plastic bottles. The fluoride solutions were agitated by a magnetic stirrer with a hot plate at room temperature. The pH was adjusted to the desired level either with 0.1 M NaOH or 0.1 M HCl. The total ionic strength adjustment buffer (TISAB) solution was added to both samples and standards in the ratio 1 : 1 in order to regulate the ionic strength of the samples and standard solutions. It was also used to avoid interferences from polyvalent cations such as Al(III), Fe(III), and Si(IV), which are able to form complex and precipitate with fluoride and reduce the free fluoride concentration in the solution. In order to determine the slope and intercept of the electrode, the fluoride ion-selective electrode was calibrated prior to each experiment. The pH meter was also calibrated at every measurement by using pH calibration buffers. All experiments were carried out at a temperature of 23 ± 2°C.

The amount of fluoride adsorbed (adsorption capacity), *q*_e_ (mg/g), at equilibrium by the adsorbent was calculated from the following expression [17]:

(1) $$\begin{array}{c} q_{\text{e}}=\frac{\left(C_{\text{o}}-C_{\text{e}}\right)}{m}\times V, \end{array}$$

where *C*_o_ and *C*_e_ are the initial and equilibrium concentrations of the fluoride solution, respectively, in mg/L, *m* is the mass of the adsorbent in *g*, and *V* is the volume of the fluoride solution in *L*.

The fluoride removal efficiency (percent fluoride removal) was calculated using the following equation [18]:

(2) $$\begin{array}{c} \text{Percent fluoride removal }\left(\%\text{ removal}\right)=\frac{\left(C_{\text{o}}-C_{\text{e}}\right)}{C_{\text{o}}}\times 100. \end{array}$$

### 2.7. Adsorption Isotherms and Kinetics

Adsorption isotherms are useful for describing how the adsorbate will interact with the adsorbent (understanding the mechanism of the adsorption) and give an idea about the theoretical maximum adsorption capacity of the adsorbent. The equilibrium data were tested for fitness into the Langmuir and Freundlich isotherm models. Langmuir isotherm [19] depends on the assumption that uptake happens on a homogenous surface by monolayer sorption without intercalation between adsorbed particles. The Freundlich adsorption isotherm is based on the equilibrium sorption on heterogeneous surfaces by multilayer sorption with interaction between adsorbed particles [20]. The linear forms of both Langmuir and Freundlich isotherm models were used to describe the adsorption capacity for a particular range of adsorbate and concentration. These linear forms were plotted for the fluoride adsorption data, and their respective isotherm constants were calculated. Adsorption kinetics is the most significant characteristic representing adsorption efficiency of the adsorbent [21]. In this study, pseudofirst-order and pseudosecond-order kinetic models were employed for understanding the kinetics of adsorption process.

## 3. Results and Discussion

### 3.1. Fourier-Transform Infrared (FT-IR) Spectroscopic Analysis of DE

**Figure 1:**
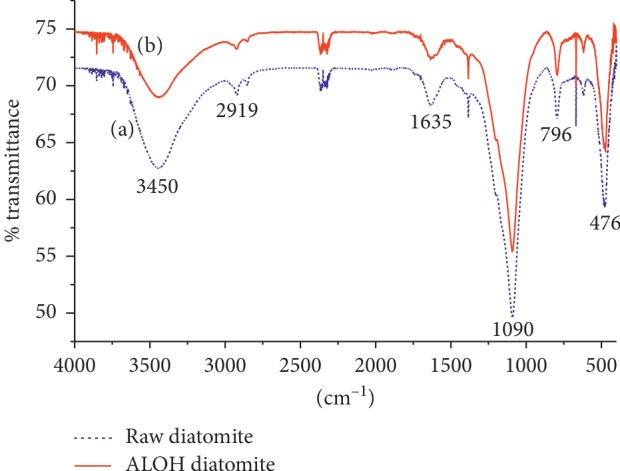
Fourier-transform infrared spectrum of raw diatomite (a) and aluminum hydroxide-treated diatomite after adsorption (b).

To understand the mechanism of fluoride-binding process on the adsorbent, the different functional groups found in the sorbent material are the key factors [22]. Hence, to identify the types of functional groups in the diatomaceous earth responsible for the fluoride uptake, an FT-IR analysis on the solid phase was carried out on the powdered adsorbent prepared in a KBr disk. The spectra of untreated (raw) diatomite and aluminum hydroxide-treated diatomite after adsorption (loading with fluoride) were measured using a Spectrum 65 FT-IR spectrometer (PerkinElmer, USA) in the range 4000–400 cm^−1^. The spectrum is shown in Figure 1.

In Figure 1, the broad peak at the 3450 cm^−1^ is due to the stretching vibration of adsorbed water-hydroxyl (O-H) and surface hydroxyl groups, and the peaks in the region 1670–1300 cm^−1^ were attributed to the bending vibrations of the adsorbed water-hydroxyl and surface hydroxyl groups. The presence of SiO_2_ is verified by absorption bands at 1090 cm^−1^ which is due to asymmetric Si-O-Si stretching vibrations and Si-O-Si bending vibrations at 476 cm^−1^. In addition, the adsorption bands at 796 cm^−1^ and 623 cm^−1^ are ascribed to Al-O absorption bands. As shown in Figure 1, there was an increase in the transmittance of the Si-OH stretching vibration at 476 cm^−1^ for the fluoride-loaded diatomite modified by treating it with aluminum hydroxide as compared to that of raw DE. This may be due to the formation of Si-F bonds with fluoride adsorption which reduced the number of Si-O-H bonds on the adsorbent surface. The same trend was observed for the transmittance at 1090 cm^−1^ for Si-O-Si stretching vibration where possible replacement of some -O-Si bonds with F^−^ could have occurred resulting in reduction of absorbance of IR by Si-O-Si. This result is in agreement with the findings of Izuagie et al. [13]. It would have been better if the interactions of the adsorbent with fluoride ions were characterized by using X-ray diffraction (XRD) and scanning electron microscopy (SEM).

### 3.2. Effects of Adsorbent Dose

**Figure 2:**
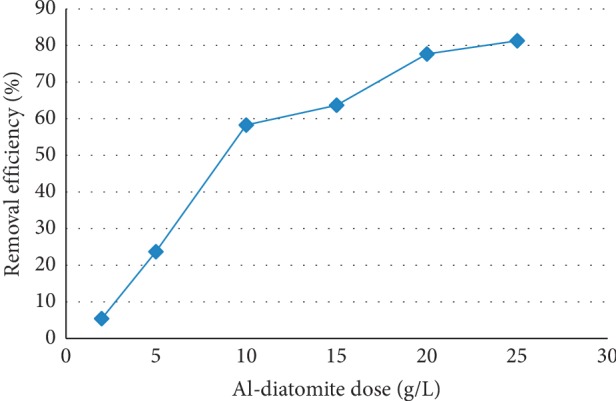
Effect of adsorbent dose on the adsorption capacity of diatomite modified by treating it with aluminum hydroxide (initial fluoride concentration 10 mg/L, pH = 6.7, contact time = 180 min, and stirring rate = 150 rpm).

The amount of contact surface between an adsorbent and adsorbate solution plays an important role in adsorption process. The effect of adsorbent mass on fluoride removal efficiency was studied by varying the mass of the adsorbent, viz., 2, 5, 10, 15, 20, and 25 g/L, while keeping other parameters constant at their respective optimum conditions (initial fluoride concentration 10 mg/L, pH = 6.7, contact time = 180 min, and stirring rate = 150 rpm). The percentage fluoride removal was determined, and the results are shown in Figure 2.

Figure 2 demonstrated that there was an increase in fluoride removal efficiency with increasing dose of the adsorbent. This is due to the increase in surface area and availability of more active sites for adsorption of fluoride [23]. Removal of excess fluoride from water by aluminum hydroxide [24] and lateritic soil [25] also revealed similar results when the adsorbent dose increases. But after a specified adsorbent dose, the percentage removal did not increase considerably and that dose was considered as optimum dose of the adsorbent. At higher dosage beyond the equilibrium, there was no appreciable increase in the percent fluoride removal due to the availability of excess adsorption sites than that of sorbents, assuming that the number of adsorption per unit mass of adsorbents remains constant. In this study, the optimum dose was found to be 25 g/L with the fluoride removal efficiency of 83.3% at an optimum pH of 6.7. The percentage removal of fluoride increased with increasing adsorbent dose, while adsorption capacity decreased gradually with dosage. The decrease in the adsorption capacity is due to the fixed initial fluoride concentration and the increased solid dose for the fixed solute load resulting in a lower availability of fluoride ions per unit mass of adsorbents. In principle, the adsorbent can be reused, regenerating it with NaOH.

### 3.3. Effect of Initial Fluoride Concentration

**Figure 3:**
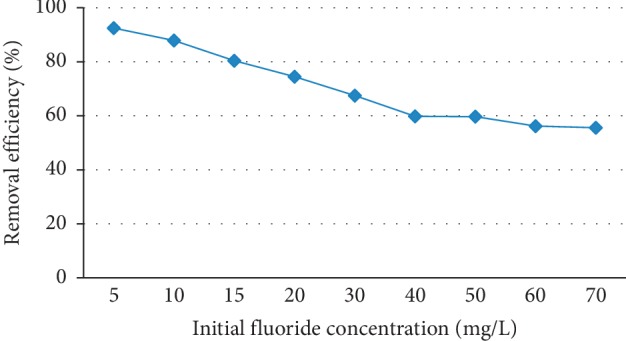
Effect of initial fluoride concentration on the adsorption capacity of diatomite modified by treating it with aluminum hydroxide (adsorbent dose = 25 g/L, contact time = 180 min, pH = 6.7, and stirring rate 150 rpm).

The effect of initial concentration on the extent of removal of the fluoride was studied by varying the concentrations from 5 to 70 mg/L, while keeping other parameters constant at their respective optimum values (pH = 6.7, contact times of 180 min, and adsorbent dose of 25 g/L). The results obtained were plotted as percentage removal of fluoride versus initial concentration of the fluoride ion in the solution as shown in Figure 3.

As can be seen from Figure 3, the percentage removal of the fluoride ion has decreased with an increase in initial concentration of the fluoride ion. This is due to saturation of the active sites of the adsorbent at higher concentrations due to the presence of more fluoride ions than the adsorption capacity of the adsorbent and the higher ratio of fluoride ions over available active surface sites with increasing initial fluoride concentration at constant mass of the adsorbent [26]. At low concentrations of the fluoride ions, sufficient numbers of active sites are available on the adsorbent, and hence, most of the fluoride ions interact with the active sites on the adsorbent; thus, percentage removal of fluoride increases until equilibrium is reached. The percentage removal (%*R*) of the fluoride ion has decreased from 92.8% to 55.6% when a fixed dose of the adsorbent used as the initial concentration of the fluoride increases from 5 to 70 mg/L. Similar studies [24, 25, 27] also pointed out that there was a decrease in fluoride removal efficiency of the adsorbents with increasing initial concentrations of fluoride.

### 3.4. Effect of pH

**Figure 4:**
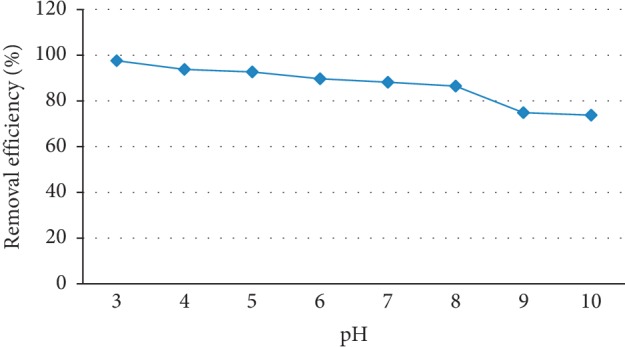
Effect of pH on the adsorption capacity of diatomite modified by treating it with aluminum hydroxide (initial fluoride concentration 10 mg/L, adsorbent dose = 25 g/L, contact time 180 min, and stirring rate = 150 rpm).

The pH of the solution is an important factor in the adsorption process since it affects the adsorbent surface properties and ionic forms of fluoride in the solution [28]. The effect of pH of the solution on the removal of the fluoride ion from an aqueous solution was studied by varying it from 3–10 while keeping other parameters constant. The pH adjustments were achieved by addition of small amounts of 0.1 M NaOH or 0.1 M HCl. Figure 4 shows the effect of pH on the fluoride removal efficiency of the adsorbent.

Most adsorbents used in fluoride removal have narrow-working pH ranges and usually show optimum performance in acidic pH range. One study indicated that maximum fluoride removal efficiencies were obtained at pH 3 for fluoride adsorption by pumice from aqueous solutions [29]. However, the pH of natural groundwater with high fluoride content is in the range of 7.6 to 8.6 in most cases [30]. Within this pH range, unlike most other defluoridation adsorbents, the adsorbent used in this study (diatomite modified by treating it with aluminum hydroxide) showed high performance, about 89% removal efficiency at 25 g/L dose and an initial fluoride concentration of 10 mg/L (Figure 4). The higher adsorption at lower pH indicates that an increase in H^+^ on the adsorbent surface makes the surface of the adsorbent positively charged resulting in electrostatic attraction between positively charged adsorbent surface and fluoride ions. The decrease in removal efficiency at higher pH values can be attributed to the competition for the active sites by OH^−^ ions and the electrostatic repulsion of anionic fluoride by the negatively charged diatomite surface. Fluoride removal above point of zero charge pH value must have been by ion-exchange as OH^−^ ions would predominate over H_3_O^+^ ions [13]. pH zero point of charge is the pH value at which the sorbent exhibits zero net electrical charge on the surface when submerged into an electrolyte.

### 3.5. Effect of Contact Time

**Figure 5:**
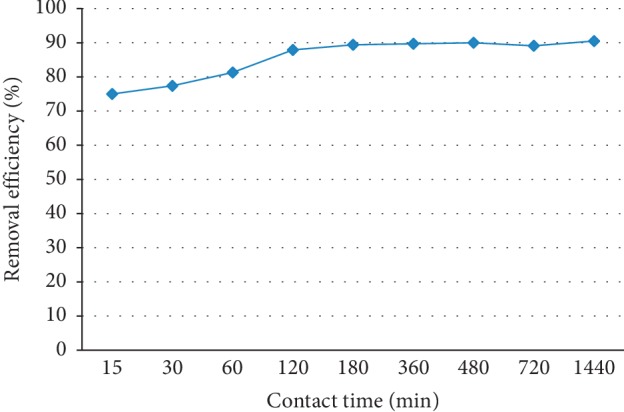
Effect of contact time on the adsorption capacity of diatomite modified by treating it with aluminum hydroxide (initial fluoride concentration = 10 mg/L, adsorbent dose = 25 g/L, pH = 6.7, and stirring rate = 150 rpm).

The study of the effect of contact time on the fluoride removal efficiency was carried out by varying it from 15 to 1440 minutes, keeping other parameters constant at optimum values (pH = 6.7, dose of adsorbent 25 g/L, and initial concentration of fluoride of solution of 10 mg/L). Figure 5 shows the effect of contact time on the defluoridation capacity of the adsorbents.

As can be seen from Figure 5, as contact time increases percent removal also increases initially and gradually attains almost an equilibrium condition in nearly 180 minutes (3 hours) and remains more or less constant thereafter. The increase was not significant for longer contact times since the reaction (defluoridation) is fast during the initial minutes. The slower adsorption at the later stage is due to a gradual diffusion of the adsorbed external fluoride to the inner adsorbent surface of the porous adsorbent for adherence, providing new fluoride binding sites on the external surface. A maximum of 89% removal could be accomplished by aluminum hydroxide-treated DE. Similar patterns were observed for removal of fluoride by rice husk [31] and removal of fluoride by using neodymium-modified chitosan [32].

Bark of babool [33], pumice [34], and trimetal-oxide adsorbent [35] have shown defloridation efficiency of 77.04, 76.64, and 78%, respectively. Thus, our adsorbent has better defluoridation efficiency compared to these adsorbents.

### 3.6. Adsorption Isotherms

An adsorption isotherm is the graphical representation of the amount of fluoride adsorbed per unit weight of the adsorbent as a function of its equilibrium concentration in the bulk solution at constant temperature. It gives general idea about the maximum amount of fluoride ions that could be removed and the effectiveness of the adsorbent in removing fluoride ions from water [36]. The Freundlich and Langmuir isotherms are the most commonly used models to investigate the adsorption processes. The Langmuir model assumes that adsorption is monolayer and is dependent on the assumption that the adsorbent surface consists of active sites having a uniform energy [19]. The Langmuir equation is written as

(3) $$\begin{array}{c} q_{\text{e}}=\frac{q_{\text{m}}K_{\text{L}}C_{\text{e}}}{1\;+\;K_{\text{L}}C_{\text{e}}}, \end{array}$$

where *q*_e_ (mg/g) is the amount of fluoride adsorbed per unit mass of the adsorbent at equilibrium, *q*_m_ (mg/g) is the maximum amount of the fluoride per unit weight of the adsorbent to form a complete monolayer on the surface, *K*_L_ (L/mg) is an adsorption equilibrium constant related to the affinity of the binding sites and energy of adsorption, and *C*_e_ (mg/L) is the equilibrium concentration of the fluoride in solution.

The linear form of Langmuir isotherm is most commonly used and is given as follows [37]:

(4) $$\begin{array}{c} \frac{1}{q_{\text{e}}}=\frac{1}{K_{\text{L}}q_{\text{m}}}\times \frac{1}{C_{\text{e}}}+\frac{1}{q_{\text{m}}}\\ \text{or }\frac{C_{\text{e}}}{q_{\text{e}}}=\frac{1}{K_{\text{L}}q_{\text{m}}}+\frac{C_{\text{e}}}{q_{\text{m}}}. \end{array}$$

The values of Langmuir constants *q*_m_ and *K*_L_ are calculated from the slope and intercept of the linear plot *C*_e_/*q*_e_ versus *C*_e_. A plot *C*_e_/*q*_e_ versus *C*_e_ should be a straight line with a slope 1/*q*_m_ and intercept as 1/*K*_L_*q*_m_. The essential feature of the Langmuir isotherm model can be expressed by means of a separation factor or equilibrium parameter (*R*_L_), which is calculated according to the following equation:

(5) $$\begin{array}{c} \text{RL}=\frac{1}{1\;+\;K_{\text{L}}C_{\text{o}}}. \end{array}$$

The values of *R*_L_ indicate the type of biosorption isotherm, and there are four possibilities for the *R*_L_ value. (i) 0 < *R*_L_ < 1 for favorable sorption, (ii) *R*_L_ > 1 for unfavorable sorption, (iii) *R*_L_ = 1 for linear sorption, and (iv) *R*_L_ = 0 for irreversible sorption.

**Figure 6:**
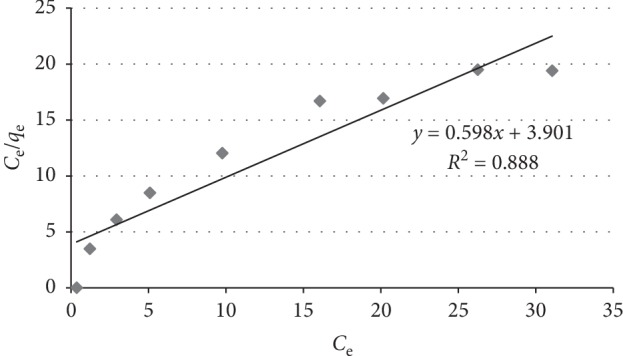
Langmuir adsorption isotherm for fluoride removal by diatomite modified by treating it with aluminum hydroxide under optimum conditions.

The linear Langmuir isothermal plot and corresponding constants are given in Figure 6 and Table 1, respectively.

**Figure 7:**
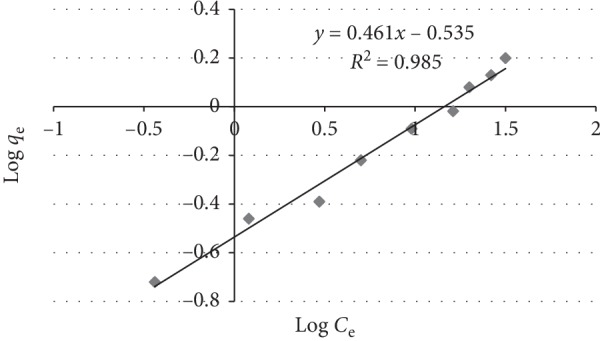
Freundlich adsorption isotherm for fluoride removal by diatomite modified by treating it with aluminum hydroxide under optimum conditions.

The plot of *C*_e_/*q*_e_ versus *C*_e_ under optimum conditions gave a straight line with a correlation coefficient (*R*^2^ = 0.888) as shown in Figure 6. The correlation coefficients are, however, less than those of Freundlich isotherm as can be seen from Figure 7.

Freundlich isotherm assumes that the uptake/adsorption of the adsorbate (fluoride) occurs on the heterogeneous surface by multilayer sorption. It is also assumed that the stronger binding sites are occupied first, and that the binding strength decreases with the increasing degree of site occupation [38]. It is given by

(6) $$\begin{array}{c} q_{\text{e}}=K_{\text{F}}C_{\text{e}}^{1/n}. \end{array}$$

The linear form of the Freundlich isotherm model is

(7) $$\begin{array}{c} \log\;q_{\text{e}}=\log\;K_{\text{F}}+\left(\frac{1}{n}\right)\log\;C_{\text{e}}\\ \text{or }\ln\;q_{\text{e}}=\ln\;K_{\text{F}}+\left(\frac{1}{n}\right)\ln\;C_{\text{e}}, \end{array}$$

where *q*_e_ (mg/g) is the equilibrium amount of fluoride adsorbed per unit mass of the adsorbent, *C*_e_ (mg/L) is the equilibrium concentration of fluoride in the bulk solution, *K*_F_ (mg/g)(mg/L)^*n*^ or (mg/g (L/mg)^1/*n*^) is a constant (Freundlich coefficient) indicative of adsorption capacity of the adsorbent, and the dimensionless constant 1/*n* indicates the intensity of the adsorption or surface heterogeneity and its value ranges between zero and one.

In general, as the *K*_F_ value increases the adsorption capacity of adsorbent for a given adsorbate increases. The plot of log *q*_e_ versus log *C*_e_ gives a straight line with a slope of 1/*n* and the intercept yields the value of log *K*_F_, indicating multilayer sorption capacity. The linear Freundlich isothermal plot and the corresponding constants are given in Figure 7 and Table 1, respectively.

The plot of log *q*_e_ versus log *C*_e_ for the sorption of fluoride on aluminum hydroxide-treated DE gave a straight line with a high correlation coefficient (*R*^2^ = 0.985) which indicates good fit of data to the Freundlich isotherm than the Langmuir isotherm. The better fitness of the data to Freundlich adsorption isotherm suggests that the adsorption of fluoride involving multilayer sorption on the surface of the adsorbent was heterogeneous. Similar results were reported on equilibrium and kinetic studies for the adsorption of fluoride onto commercial activated carbons [39]. Table 1 presents the calculated parameters for Langmuir and Freundlich isotherms.

Compared with the Langmuir isotherm (*R*^2^ = 0.888), the Freundlich isotherm (*R*^2^ = 0.985) model was better fitted to describe the adsorption characteristics of fluoride on aluminum hydroxide modified with diatomite. The value of 1/*n* (0.461) lying between 0.1 and 1.0 and that of *n* (2.17) lying in the range 1–10 both confirmed the high bond strength between the adsorbate and the adsorbent, and it also confirmed the adsorbent surface was heterogeneous. Furthermore, the heterogeneity of the adsorbent surface is established by the low values of the parameter 1/*n*. The small value for the magnitude of the Langmuir constant “*b*” (0.2 L/mg) indicates a low heat of adsorption [13]. The *R*_L_ value (0.44) lying between 0 and 1 indicated favorable conditions for sorption. The values of *q*_m_ obtained from the Langmuir model is 1.67 mg/g.

### 3.7. Adsorption Kinetics

Kinetic modeling gives information about adsorption mechanisms and possible rate-controlling steps such as mass transport or chemical reaction processes. The adsorption rate is an important factor for a better choice of material to be used as an adsorbent, where the adsorbent should have a large adsorption capacity and a fast adsorption rate. Pseudofirst-order and pseudosecond-order models were used to study the adsorption kinetics. For the pseudofirst-order model, the adsorption rate is expected to be proportional to the first power of concentration, where the adsorption was characterized by diffusion through a boundary [40]. For the pseudofirst-order model under initial and end boundary conditions *t* = 0 to *t* = *t* and *q*_t_ = 0 to *q*_t_ = *q*_t_, a linear equation is obtained as follows:

(8) $$\begin{array}{c} \log\left(q_{\text{e}}-q_{\text{t}}\right)=\log\;q_{\text{e}}-\frac{k_{1}}{2.303}t\\ \text{or }\ln\left(q_{\text{e}}-q_{\text{t}}\right)=\ln\;q_{\text{e}}-k_{1}t, \end{array}$$

where *q*_e_ and *q*_t_ are the amounts of solute adsorbed at equilibrium and at a given time *t*, respectively and *k*_1_ is the first-order rate constant. A plot of “ln (*q*_e_ − *q*_t_)” versus “t” gives a straight line with an intercept of “ln *q*_e_” and slope of “−k_1_.”

**Figure 8:**
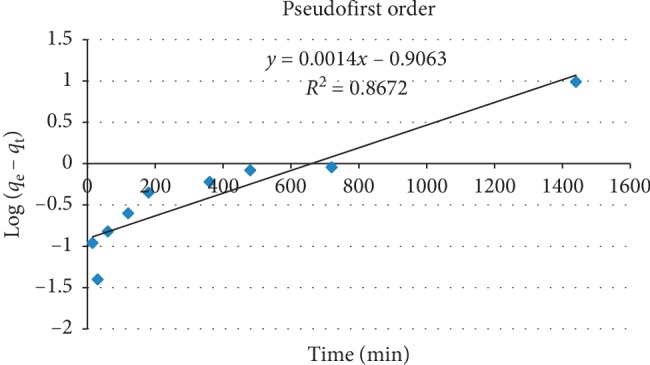
Pseudofirst-order plot for kinetic data for fluoride removal by diatomite modified by treating it with aluminum hydroxide under optimum conditions.

The plot for pseudofirst-order is given in Figure 8.

The pseudosecond-order model assumes that chemisorption may be the rate-controlling step in the adsorption processes. For the pseudosecond-order model under the initial and end boundary conditions *t* = 0 to *t* = *t* and *q*_t_ = 0 to *q*_t_ = *q*_t_, a linear equation is obtained [40] as follows:

(9) $$\begin{array}{c} \frac{t}{q_{\text{t}}}=\frac{1}{k_{2}q_{\text{e}}^{2}}+\frac{1}{q_{\text{e}}}t. \end{array}$$

The equilibrium adsorption capacity (*q*_e_) and the second-order constant *k*_2_ (g/mg·h) can be determined experimentally from the slope and intercept of plot t/*q*_t_ versus *t*. A plot of t/*q*_t_ against *t* gives a straight line with an intercept of 1/*k*_2_*q*^2^_e_ and slope of 1/*q*_e_.

**Figure 9:**
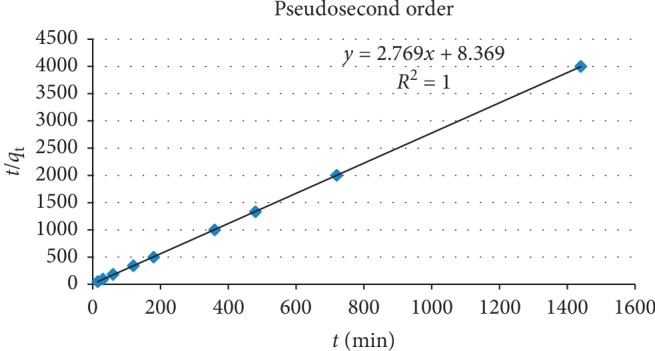
Pseudosecond-order plot for kinetic data for fluoride removal by diatomite modified by treating it with aluminum hydroxide under optimum conditions.

The plot for pseudosecond-order is given in Figure 9.

Table 2 shows the values of pseudofirst-order and pseudosecond-order kinetic constants and intraparticle diffusion model.

The value of correlation coefficient (*R*^2^ = 1) is high for the pseudosecond-order kinetic model compared to that of pseudofirst-order (*R*^2^ = 0.867), and this indicates that the experimental data of the present study best fits to the pseudosecond-order model [41]. The value of *k*_2_ is high which indicates the tested adsorbent is effective in fluoride adsorption [26].

### 3.8. Adsorption Mechanisms

The probable mechanism controlling the sorption rate was evaluated using the intraparticle diffusion model. The McKay and Poots equation is expressed as [42]

(10) $$\begin{array}{c} q_{\text{t}}=k_{\text{id}}t^{1/2}+I, \end{array}$$

where *q*_t_ is the amount of fluoride adsorbed (mg/g) at time *t* (min), *k*_id_ is the intraparticle diffusion rate constant (mg/g·min^1/2^), and *I* (mg/g) is a constant that has to do with the thickness of the boundary layer.

**Figure 10:**
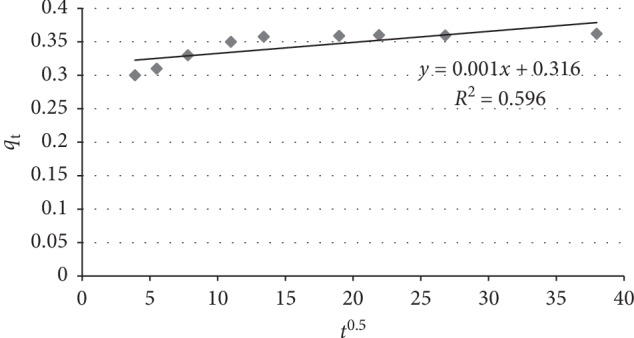
Intraparticle diffusion kinetic plot for fluoride removal using diatomite modified by treating it with aluminum hydroxide.

According to the model, plot of uptake, *q*_t_ versus square root of time (*t*^1/2^) should give a linear line if intraparticle diffusion is involved in the sorption process. *k*_id_ and *I* values are obtained from the slopes and intercept of the linear plot, respectively. If the plot of *q*_t_ against *t*^1/2^ is linear, intraparticle diffusion would be the rate-determining step. The low correlation coefficient value of *R*^2^ = 0.596 for the intraparticle diffusion model (Table 2) and the fact that linear portions of the curves do not pass through the origin (Figure 10) indicate that the intraparticle diffusion model is not the rate-determining step of the adsorption process. Intraparticle diffusion could possibly not be the sorption rate determinant because of the much smaller size of fluoride ions to the pores of the sorbent. The ionic radius of fluoride is 1.33 A^o^ [13].

Thus, the probable mechanism controlling the rate of fluoride sorption onto the adsorbent is either the electrostatic attraction of fluoride ions to the positively charged sorbent surface or the ion-exchange at the surface [28, 43]. If the data exhibit multilinear plots, then two or more steps influence the sorption process. In general, a mass transfer process is diffusion controlled and its rate is dependent upon the rate at which components diffuse towards one another [44].

### 3.9. Application to Real (Field) Drinking Water Samples

**Figure 11:**
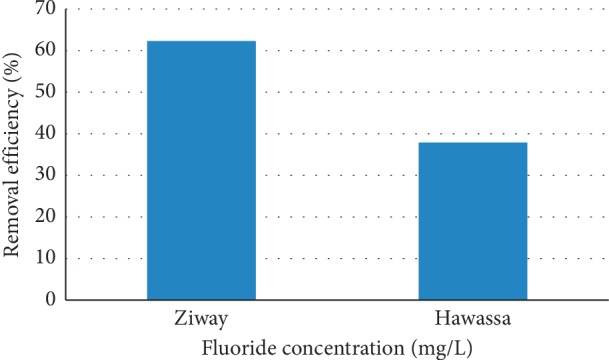
Application of fluoride removal from the Rift Valley water samples using diatomite modified by treating it with aluminum hydroxide (adsorbent dose 25 g/L; contact time 180 minutes).

The performance of aluminum hydroxide-treated DE was evaluated by applying it for defluoridation of real groundwater samples taken from Hawassa and Ziway, having 13.2 and 7.5 mg/L of initial fluoride concentrations, respectively. These are the areas located in the Rift Valley of Ethiopia having high fluoride concentrations in water. The defluoridations of real water samples were done at optimum experimental conditions, and the results obtained are presented in Figure 11.

As can be seen from Figure 11, the percentage removal of fluoride from real groundwater samples taken from Ziway and Hawassa is about 60% and 40%, respectively. The results in Figure 11 indicate that fluoride removal efficiency of the aluminum hydroxide-treated DE from the groundwater samples was lower than that of the synthetic simulated NaF solutions. Most of the groundwater samples taken from the Ethiopian Rift Valley contained high concentration of bicarbonate (HCO_3_^−^), sulfate (SO_4_^2−^), and other anions [45]. The pH of the real Ziway and Hawassa groundwater samples was measured to be 8.97 and 8.31, respectively. In the basic solution, the presence of high concentration of HCO_3_^−^ and SO_4_^2−^ ions interfere the uptake of fluoride ions by the adsorbents [46].

## 4. Conclusions

Diatomite modified by treating it with aluminum hydroxide was found to be an effective adsorbent for the defluoridation of aqueous solution and natural groundwater. The maximum percent ﬂuoride removal and adsorption capacity were 89% and 1.67 mg/g, respectively, for 10 mg/L ﬂuoride-spiked water under optimum adsorption conditions (contact time: 180 min, adsorbent dosage: 25 g/L, pH 6.7, at room temperature, and shaking speed: 150 rpm). The adsorption data fitted well with Freundlich adsorption isotherm with a good correlation coefficient value which indicates multilayer sorption on the heterogeneous adsorbent surface. Sorption kinetics was studied by using pseudofirst-order and pseudosecond-order kinetic models. The data fitted better to pseudosecond-order kinetics which showed that the adsorption was by chemisorptions. Since intraparticle diffusion was not the rate-limiting mechanism, the adsorption rate-limiting step was most probably the process involving ion-exchange or attraction of F^−^ to the sorbent surface. The results of the study showed that this low-cost adsorbent material, DE, can be employed for fluoride removal from groundwater and other water samples which contain excessive amount of fluoride which could be detrimental to human health. This adsorbent is cheaper, abundant, and easily available in huge amount in Ethiopia.

## Figures and Tables

**Table 1:** Calculated Langmuir and Freundlich isotherm parameters.

Calculated Freundlich isotherm constants			Calculated Langmuir isotherm constants			
*K*_F_	1/*n*	*R* ^2^	*R*_L_	*b* (L/mg)	*R* ^2^	*q*_m_ (mg/L)
0.29	0.461	0.985	0.44	0.2	0.888	1.67

**Table 2:** Pseudofirst-order and pseudosecond-order kinetic constants and intraparticle diffusion model parameters.

Pseudofirst order			Pseudosecond order			Intraparticle diffusion model parameters	
*k*_1_	*q*_e_	*R* ^2^	*q*_e_	*k*_2_	*R* ^2^	*k*_id_	*R* ^2^
0.0023	0.124	0.867	0.36	0.92	1	0.001	0.596

## Data Availability

The data used to support the results of this study are included within the article, and any further information is available from the corresponding author upon request.
